# Knowledge translation in Uganda: a qualitative study of Ugandan midwives’ and managers’ perceived relevance of the sub-elements of the context cornerstone in the PARIHS framework

**DOI:** 10.1186/1748-5908-7-117

**Published:** 2012-12-03

**Authors:** Anna Bergström, Stefan Peterson, Sarah Namusoko, Peter Waiswa, Lars Wallin

**Affiliations:** 1 Department of Public Health Sciences, Division of Global Health (IHCAR), Karolinska Institutet, Nobels väg 9, Stockholm SE-17177, Sweden; 2 Department of Women’s and Children’s Health, International Maternal and Child Health (IMCH), Uppsala University, Akademiska sjukhuset, Uppsala SE-75185, Sweden; 3 School of Public Health, Makerere University College of Health Sciences, Plot 1, New Mulago Hospital Complex, Makerere, Uganda; 4 Department of Neurobiology, Care Sciences and Society, Division of Nursing, Karolinska Institutet, Alfred Nobels allé 23, Huddinge, 141 83, Sweden; 5 School of Health and Social Studies, Dalarna University, Falun, SE-79188, Sweden

**Keywords:** Organizational context, PARIHS, Knowledge translation, Low-income settings, Focus group discussions, Interviews

## Abstract

**Background:**

A large proportion of the annual 3.3 million neonatal deaths could be averted if there was a high uptake of basic evidence-based practices. In order to overcome this ‘know-do’ gap, there is an urgent need for in-depth understanding of knowledge translation (KT). A major factor to consider in the successful translation of knowledge into practice is the influence of organizational context. A theoretical framework highlighting this process is Promoting Action on Research Implementation in Health Services (PARIHS). However, research linked to this framework has almost exclusively been conducted in high-income countries. Therefore, the objective of this study was to examine the perceived relevance of the sub-elements of the organizational context cornerstone of the PARIHS framework, and also whether other factors in the organizational context were perceived to influence KT in a specific low-income setting.

**Methods:**

This qualitative study was conducted in a district of Uganda, where focus group discussions and semi-structured interviews were conducted with midwives (n = 18) and managers (n = 5) within the catchment area of the general hospital. The interview guide was developed based on the context sub-elements in the PARIHS framework (receptive context, culture, leadership, and evaluation). Interviews were transcribed verbatim, followed by directed content analysis of the data.

**Results:**

The sub-elements of organizational context in the PARIHS framework—*i*.*e*., receptive context, culture, leadership, and evaluation—also appear to be relevant in a low-income setting like Uganda, but there are additional factors to consider. Access to resources, commitment and informal payment, and community involvement were all perceived to play important roles for successful KT.

**Conclusions:**

In further development of the context assessment tool, assessing factors for successful implementation of evidence in low-income settings—resources, community involvement, and commitment and informal payment—should be considered for inclusion. For low-income settings, resources are of significant importance, and might be considered as a separate sub-element of the PARIHS framework as a whole.

## Background

Translating knowledge into practice has been shown to be a slow and nonlinear process [1]. The importance of knowledge translation (KT) is its potential to bridge the gap between what is known and what gets done in practice, also called the ‘know-do’ gap [2]. One striking example of the global ‘know-do’ gap is the estimate that up to 70% of neonatal deaths could be averted with higher levels of implementation of basic and predominately cost-effective evidence-based practices (EBPs) [3,4]. These interventions cover the antepartum, intrapartum, and postpartum period, and include measures such as immediate breastfeeding, prevention and management of hypothermia, and kangaroo mother care for low-birthweight newborns. However, research on KT originates mainly from high-income countries, leaving the settings where 99% of the annual 3.3 million neonatal deaths occur with scarce empirical knowledge on how to translate evidence into routine practice [5,6].

In order to understand the effectiveness of KT interventions, efforts should be directed at considering the influence of contextual factors [7]. There are several theories, models, and frameworks that emphasize the importance of context in KT, and evidence affirms its importance ([8,9]). The importance of better documentation and understanding of context in low-income countries has been repeatedly emphasized [10-14]. McCoy *et al*. [15] claim that lack of sensitivity to context and the socio-political nature of health systems partly explain the frequent failure to bridge the ‘know-do’ gap. Better mapping of context has also been found to improve implementation by allowing for strategic tailoring of implementation strategies [16] and by providing opportunities to interpret findings in KT intervention studies.

The’Promoting Action on Research Implementation in Health Services’ (PARIHS) framework argues that there are three interacting cornerstones that positively influence KT: strong evidence, supportive organizational context, and appropriate facilitation [17]. Since the first PARIHS publication in 1998, the framework has been subject to evaluation, which has provided reasonable evidence for validity of its content and constructs [18-22]. Presently, the framework is also being evaluated in large-scale studies in both Europe and Vietnam [23,24]. Furthermore, the framework was recently subjected to systematic critical synthesis where authors concluded that there is empirical support for the separate cornerstones, although there is a need for rigorous prospective studies where the framework is used and evaluated [25]. The extensive use and ongoing evaluation of the framework presented an appropriate base for the work reported in this paper.

**Figure 1:**
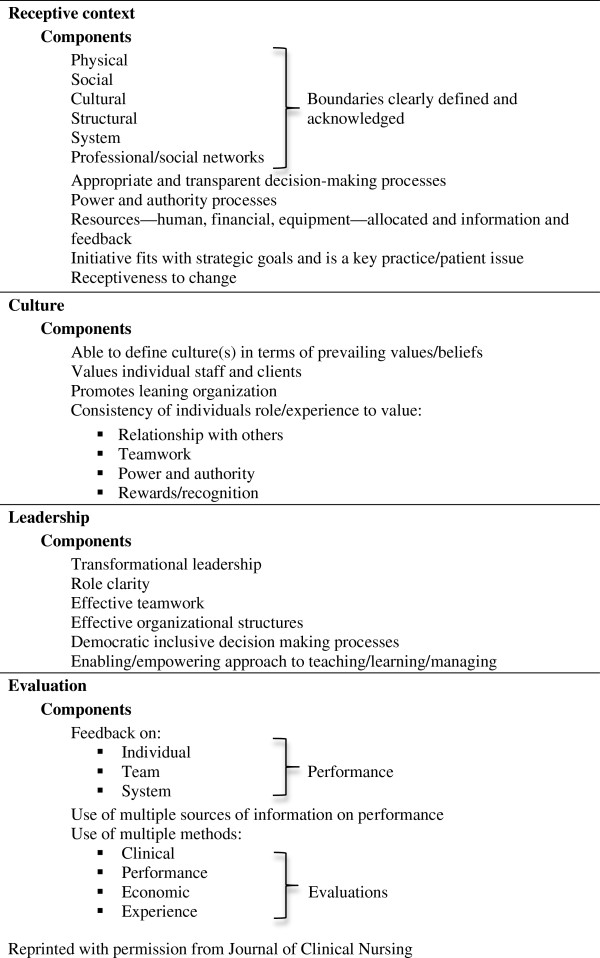
**The four sub elements of the** ‘**context**’ **cornerstone in the PARIHS framework [**[22]**].**

The second PARIHS cornerstone is defined as ‘the environment or setting in which the proposed change is to be implemented’ and includes four sub-elements: receptive context, culture, leadership, and evaluation [22]. Receptive context includes structural and resource related aspects of context. The culture sub-element proposes that organizational cultures that can be described as ‘learning organizations’ are more conductive to change. Culture has both been regarded as something an organization *is* as well as something that the organization *has*. When considering culture as something the organization *has*, the organization is viewed as comprised of several characteristics that can be isolated, described and manipulated [26]. Organizational culture, seen from the *is* perspective, has been described as the ‘glue’ that holds an organization together and stimulates the employee’s commitment [27]. The authors to the PARIHS framework has described culture as degrees of clarity in values and beliefs, the level of regard for individuals, the organizational ‘drive’ (task versus learning), the degree of consistency in valuing relationships, teamwork, power, and authority, and the extent of recognition or reward that is provided [20] or, simply put ‘the way we do things’ [20]. Leadership summarizes the nature of human relationships in the practice context. Leaders play a key role in creating ‘learning organizations.’ The PARIHS framework claims that transformational leaders, as opposed to autocratic leaders, have the ability to challenge individuals in an inspiring and enabling way [20]. Transformational leaders ‘articulate a vision or mission and challenge their followers by providing a personal example’ and ‘have the ability to commit themselves and allow others to optimize their skills, abilities, knowledge, and potential’ [28,29]. The last sub-element, evaluation, highlights contexts in which feedback based on organizational and individual evaluation is performance on a regularly basis. Evaluation has, in the field of public health been defined as ‘efforts aimed at determining as systematically and objectively as possible, the effectiveness and impact of health-related (and other) activities in relation to objectives and taking into account the resources and facilities that have been deployed in the activities being evaluated’ [30]. Evaluation, according to the PARIHS framework, is primarily comprised of the utilization of locally derived data [20]. McCormack *et al*. claim that effective healthcare cultures use evidence gathered from several different sources to support decisions about performance of individuals and the organization [20]. Features of context according to the PARIHS framework are elaborated upon in Figure 1[22].

Recently, three quantitative instruments have been developed to assess context aspects of the PARIHS framework [31-33]. The Alberta Context Tool consists of eight dimensions: leadership, culture, evaluation, social capital, formal interactions, informal interactions, structural and electronic resources, and organizational slack with three separate concepts—staffing, space, and time [32]. The Organizational Readiness to Change Assessment is developed to asses the whole PARIHS framework, its context cornerstone is assessed with five dimensions: culture, leadership, measurement and readiness for change, and resources. Lastly, the Context Assessment Index includes five constructs: collaborative practice, evidence-informed practice, respect for persons, practice boundaries, and evaluation. All the three tools were developed for high-income settings and their psychometric properties are presently being investigated in several studies. Research based on PARIHS has almost exclusively been conducted in high-income countries [25], and it is not known whether its cornerstones and sub-elements are also of relevance for KT in low-income settings, or whether other aspects of context are at play. Therefore, inspired by the three context tools developed from PARIHS and, as a first step towards developing an instrument to assess organizational context in low-income settings, the objective of this study was to examine the perceived relevance of the sub-elements of the organizational context cornerstone of the PARIHS framework, and whether additional factors in the organizational context were perceived to influence KT in Uganda.

## Methods

### Study setting and design

This study was carried out in a district of Uganda with about 20 health centers providing delivery services, including one general hospital with a bed capacity of about 100. The hospital has a catchment area beyond the district limits, and serves about 1.5 million individuals. The majority of people in the district earn their livelihood through farming. The study was conducted within a larger study with the aim to develop a quantitative assessment tool regarding context in low- and middle-income settings. The larger study is conducted within the Research for Improved Child Health network, and efforts similar to the study reported here are undertaken in Vietnam and Bangladesh; findings from those studies will be reported elsewhere. This study was carried out in a district where efforts to improve neonatal health and survival was ongoing, subjecting health workers, primarily midwives, and managers to change.

### Data collection

A semi-structured guide was developed based on the four sub-elements of the context cornerstone (receptive context, culture, leadership, and evaluation) as suggested in the PARIHS framework (Figure 1) and inspired by the dimensions within its three developed tools [22,31-33]. Focus group discussions (FGDs) and individual interviews were conducted with midwives working in different levels of the healthcare services in the district in 2010. Individual interviews were also conducted with managers, for example, those in charge of health centers and health service managers at district level. All FGDs and individual interviews were conducted outside the respondent’s place of work to ensure confidentiality and allow for an open discussion. FGDs are considered a useful method for exploring new areas, because the interaction among group members brings out different opinions about the topic under discussion [34]. It has also been suggested that FGDs are a good data collection technique when discussing sensitive topics [35]. In this study, the FGDs served well for exploring prevailing perceptions about organizational context among midwives working at different health centers. However, they were less helpful when conducted with midwives working within the same unit, because it was challenging for participants to discuss leadership. Therefore, we conducted individual interviews with midwives working in the same unit. During the FGDs and interviews, the interviewers tried to clarify unclear concepts, and summarized the respondents’ statements to ensure clarity. To ensure credibility of our study, we triangulated methods as described above. Triangulation of methods allowed for the exploration of different aspects of the study objectives. Respondents were provided with reimbursement for their transportation costs.

Following a pilot FGD with Ugandan midwives, to ensure comprehensiveness of the guide, the guide was used in both FGDs and individual interviews (Additional file 1). At the beginning of each session, respondents were asked to think of and briefly describe how the introduction of new knowledge and change in practice had occurred in their place of work, and throughout the session try to attach their perceptions of the relevance of the organizational context to those changes. In relation to the ongoing intervention to improve neonatal health and survival, several such changes were brought up during discussions, for example, neonatal resuscitation according to guidelines, the utilization of incubators, and the introduction of death review meetings.

Data collection sessions were conducted in English (Uganda’s official language) and audio-recorded. Sessions lasted 45–110 minutes and were performed by AB and SN. After each data collection session, AB and SN discussed what had emerged, whether any changes should be made to the guide, and whether further probes were needed.

### Participants

We conducted two FGDs and a total of 10 individual interviews. All respondents were given written information about the study and agreed to participate. Two FGDs were conducted: one with six midwives from community health centers and one with midwives working in the hospital. Sampling for the first FGDs was purposive, whereby respondents from different parts of the district, working under different conditions in terms of distance to the district hospital and number of healthcare workers in the unit, were included. The second FGD included seven conveniently sampled midwives working in the antenatal clinic at the hospital. The reason for choosing this division was that the organizational context differed between the primary healthcare units and the district hospital. Because some aspects of the interview guide, primarily leadership, were difficult to discuss during the FGDs, the study team opted to continue data collection by conducting individual interviews with other midwives working in the same unit.

Sampling for individual interviews with midwives and managers employed a purposive snowballing method [36]. In total, 23 (22 female, 1 male) individuals participated in the study; the mean age was 39 years (range, 26–55 years), the median years since qualification was eight (range, 2–34), and the median number of years they had worked in the present place of work was four (range, 1–30 years) (Table 1). The reason for inviting midwives and managers involved in the provision of maternal and neonatal health and survival was the fact that there was an ongoing intervention study in the district from which participants could draw experiences.

**Table 1:** Description of participants

**Data collection method**	**Participants**	**Place of work**
FGD I	Six female midwives.	Lower-level HCs.
FGD II	Seven female midwives.	The antenatal clinic at the hospital.
Individual interviews with midwives	Five female midwives.	The maternity ward at the hospital.
Individual interviews with managers	Five managers:	Two of the midwives and the clinical officer worked at district health office whereas the third midwife and the physician worked at the hospital.
	· Three female midwives	
	· One female clinical officer	
	· One male physician.	

### Data analysis

Preliminary analysis and discussions were held directly after each FGD and interview to agree on the level of saturation, that is, when the researcher is no longer hearing new information and ends data collection. The audio-recorded data were transcribed verbatim by AB and imported to QSR NVivo 8 software, followed by primarily using directed content analysis as suggested by Hsieh and Shannon [37]. The goal of a directed content analysis is to validate or conceptually extend a theoretical framework or theory [37]. This deductive directed approach implied a more structured process compared with inductive content analysis. Using prior research and existing theory, in this case the PARIHS framework and publications relating to it [17,18,20-22,25], a thorough reading of the transcripts was followed by identifying and highlighting key concepts that represented the four sub-elements in the semi-structured guide. Next, all highlighted passages were coded. Further reading, and employing an inductive approach, as suggested by Graneheim and Lundman [38], led to the identification of additional factors perceived to impact upon the implementation process, which could not be categorized within the initial scheme. AB performed the analysis and findings were then discussed in the research group to reach consensus with regard to what they reflected. Examples of the analysis process are presented in Tables 2 and 3. In addition, we discussed our findings with peer de-briefers to provide a fresh perspective for analysis and critique [39]. In this study, peer de-briefers included two health practitioners and public health researchers from low-income settings and one Swedish implementation researcher. In total, we involved three peer de-briefers to question the findings from their separate perspectives. 

**Table 2:** Example of the qualitative directed content analysis process

**Theme**	**Meaning unit**	**Condensed meaning unit**	**Category**
Evaluation	‘We could also ask questions concerning that particular patient. Even other patients. If you have a knowledge gap you ask the doctor and from there he will also tell you what you are really supposed to do, then you pick up from there.’	Audit meetings helps the team in identifying gaps in practice	Evaluating provided healthcare can lead to improvements
	‘You know that if we had more of such meeting we would go on improving. Gradually. If we in another meeting raise another problem it also gets solved.’	Meetings facilitates solving problems	
	‘And then everybody reacts on those comments and you look forward, what are we going to do?’	Meetings assisting team to improve	
	‘And in most cases now when we go for these maternal audits and what, that perinatal audit. When you have sat in that meeting and you see what ever had happened to that patient and you see that whatever was supposed to happen to that patient was not done. That’s when you realize there is a gap’	Audit meetings helps the individual in identifying and acting on knowledge and practice gaps	
	‘They [evaluation tools] come from the Ministry of Health, they are Ministry of Health checklists, but it is quite a big book eh? So it depends on what you will check on, on that particular day.’	Support supervision is undertaken utilizing tools from Ministry of Health.	Evaluating practices on-site is key
	‘If you give the skills and knowledge to the participants and then you don’t conduct supportive supervision, at times they might not implement. So, supportive supervision is the key component in ensuring that health workers do practice the new skills and knowledge.’	Support supervision is a key component in ensuring that health workers practice their new skills and knowledge.	
	‘You go in a health facility and you sit, that whole day you go and sit with that person and you look at her and you see how she does it and then you sit with her and work with her and says ‘that this is how you should have done it, this is how you should fill the register, this is how you should order your antiretroviral drugs.’	Support supervision requires observing how health workers do things	

**Table 3:** Example of the qualitative inductive analysis process

**Meaning unit**	**Condensed meaning unit**	**Category**	**Theme**
‘Drugs disappear, because we are poor, they have to eat! Should they [*health workers*] go and steal on the street? So, that is how people are surviving.’	Selling drugs to survive	Informal payments is a coping strategy	Informal payment
‘Me I feel that because of that very little funding people have continued to request money from the patients.’	Low salaries fueling under table payment		
‘If I have paid to get my job, then I’ll work without earning salary, how am I going to survive? I have to get a way of surviving, either I’ll sell the service or I’ll sell the drugs of the hospital.’	Paying for jobs fueling under table payment		
‘So Sister X is invited to the committee to ask the technical questions, after that, she goes out. She is not invited for the evaluation; it is for that committee to decide who is to get that job. So it is than that the committee says ‘we want such and such of money.”	Acquiring a position not only based on who has the best technical knowledge	Informal payments might lead to lack of competences	
‘Even in the trainings I understand, they tell you that, ‘for us we came to pass eh, because we paid our money.’ So, at times, the basic knowledge they have, is not adequate.’	Lacking basic knowledge because it is possible to pay to pass		

### Ethics

Ethical approval was obtained from the Makerere University School of Public Health Review Board and the Uganda National Council of Science and Technology. All respondents were given written information about the study prior to participation and written consent was obtained. Voluntary participation and confidentiality were ensured, and respondents were informed of their right to withdraw from the study at any time. They were also told that data would be analyzed after being de-identified. Data collection was undertaken outside of respondents’ working units to ensure confidentiality and avoid disturbance.

## Results

In addition to the PARIHS sub-elements, ‘commitment and informal payment’ emerged as one additional contextual factor within the inductive analysis. Findings are presented under the headings of the factors identified in the current study and the four sub-elements of the PARIHS framework.

### Commitment and informal payment

The individual health workers’ commitment to their work was brought up as a major aspect of how context influences the implementation of new practices. This element was commonly referred to as ‘loss of morale’ due to scarce resources, low salaries, little appreciation, a heavy workload, and the presence of informal payment:

‘… Though I know that salary, or money is not a motivator … but if it’s not there it is a demotivator! … Without proper salary, people come to work for the sake of being on duty. They end up coming late. They come late and leave early. So, the factor here is called demotivation. People are demotivated. So, even when you teach them something new, they are reluctant to take it up.’ [Manager, individual interview]

In individual interviews with midwives and managers, the problem of informal payment emerged, such as patients having to pay for drugs and services that should be available for free:

‘We have to get a way of surviving, either sell the service or sell the drugs of the hospital. Drugs disappear, because we are poor … that is how people are surviving.’ [Manager, individual interview]

Another type of informal payment that interviewees were familiar with occurred during the employment process. It was described that it was common to pay to get a position. One issue raised in relation to this practice was that it might not be the person best suited for the job who was offered the position, but rather the one who paid the most for it.

### Receptive context

When broadly discussing what influenced KT, respondents brought up ‘resources’ as an issue that influenced both the implementation of new knowledge and healthcare services overall. In the term ‘resources,’ respondents included human resources, equipment, drugs and supplies, space, means of transport, and time. As an example, respondents expressed frustration over their experience of coming back from training to their place of work and failing to be able to implement new skills due to lack of resources. A respondent who had recently attended a course in infection prevention and control highlighted the impact of lack of resources:

‘… that week we had a problem, it started with lack of water, then eventually lack of soap, we could not sterilize our equipment. We had emergencies. Imagine, how do you repair a ruptured uterus? Instruments are there but you don’t have linen! You don’t have linen, sterilized linen. We ended up, however, improvising. But that week we had a lot of sepsis on the ward. A lot!’ [Manager, individual interview]

### Culture

Respondents considered on-the-job learning from peers as one of the most important ways to acquire knowledge on best practices. Midwives described how this occurred when they were faced with new challenges and needed help to cope with the situation. Study participants commonly raised the necessity of good communication and cooperation among the health workers in the unit. In fact, teamwork was perceived as more important than many other KT efforts (such as training) in order for new practices to become routine:

‘One thing that hinders implementation is poor communication among ourselves. If we are going to implement A, B, C, D, but we’re not cooperating, the thing is not going to move.’ [Midwife, individual interview]

The intraprofessional teamwork was generally considered as supportive, whereas the lack of trust and teamwork between different professions was brought up as an obstacle for providing high-quality care. Respondents also expressed that ‘fear of being accused of doing wrong’ was overriding the trust in the greater team and its ability to work out problems. When discussing culture, midwives from the hospital told how lower-level health workers feared expressing their views in meetings with different cadres of healthcare providers. Respondents viewed these meetings as components of the organizational structure that could potentially play a larger role in translating knowledge into practice, if the culture changed to allow the engagement of all health workers in discussing service delivery.

### Leadership

Midwives working in the hospital saw the leader as ‘one of themselves’ rather than as superior to them. In contrast, midwives working in lower-level health centers expressed frustration at working under unclear leadership, stating that the leader was neither present nor part of the team. The perceived importance of having a capable leader for KT was clear, whereby the leader was seen as a person that should be part of the working team and while also acting as a role model. Respondents also believed a good leader should inspire and support professional development. Leaders in the hospital were perceived as being physically present and open to inviting staff to participate in organized continuing medical education meetings. In contrast, midwives working in lower-level health centers in isolated rural areas were not often invited for short courses or continuing medical education meetings, and instead relied on their leader for the provision of new knowledge, which further illuminates the importance of leadership in that setting.

Although the leader in the hospital unit was seen as part of the team, midwives feared this higher leadership. The strong hierarchical structure was highlighted as informants used words like ‘autocratic leadership’ to describe the absence of teamwork for meeting challenges faced by the organization:

‘… the big man will call you, sometimes when the patient is there with the attendant and ask, ‘Why haven’t you given treatment? You want this patient to die?!’ These things are discouraging. You know, an autocratic leadership style? People get demotivated. They just do something because they fear, they work under pressure, they work under tension.’ [Manager, individual interview]

### Evaluation

The perceived importance of supportive supervision and formal meetings to discuss how to overcome adverse outcomes was clear. On an individual level, the importance of supportive supervision by a superior, both as a way of detecting gaps as well as to directly correct faults, was discussed. At the unit level, midwives perceived that a functioning system to evaluate the health services provided was crucial—both to identify existing gaps and to monitor the implementation of new interventions.

One strategy considered to be effective in recognizing ‘know-do’ gaps was the routine death review meetings with an audit component. In these meetings, team members would systematically share anything they knew regarding the circumstances of the death of a patient, followed by discussion of possible avoidable causes or malpractices. Further, the team tried to come up with solutions to the malpractices identified. This initiative had recently been introduced with the aim of reducing the maternal and perinatal mortality in the hospital, and was perceived as a promising way for the unit to identify knowledge gaps and bring about change. However, although respondents found these meetings useful, they also expressed the need to improve the quality of meeting documentation in order to improve the quality of feedback data and the subsequent actions taken on the basis of such data.

In general, respondents appreciated evaluation at unit level, whereas opinions about individual evaluation, feedback, and recognition were mixed. Some respondents felt that individual feedback was important in order to address gaps in their knowledge and skills. It was also stated that positive feedback given to one person influences others to ‘aim higher.’ However, others mentioned that recognized high-quality performers would, out of jealousy, be ‘punished’ by being given an increased workload by colleagues. To avoid biased appraisals, it was suggested that standards should be developed for the appraisal procedure. Respondents also asked for tangible criteria towards which they could strive.

In addition to the evaluation and feedback occurring within the healthcare organization, respondents also underlined the importance of community and client feedback when asked how knowledge and practice gaps were identified and why change occurs. In particular, midwives working in primary healthcare centers brought up community involvement as a driving force for change. Midwives received continuous feedback from the community on both negative and positive aspects of the healthcare services. In some communities, the community chairman was in constant dialogue with the health workers and gave regular feedback on perceived improvements as well as negative incidents. Community engagement was described as a growing demand from the community for access to health services and for improved health services. Furthermore, community members were perceived as ‘inquisitive’ as demonstrated in the following quotation.

"‘So, when you come back and they see no change, they say, ‘Now what type of training was that? Did they really train her? I think maybe she was not trained well.’ You see, these community members, they are like that. But if they see a change, say better care of the newborn, then they appreciate.’ [Midwife, FGD]"

## Discussion

**Figure 2:**
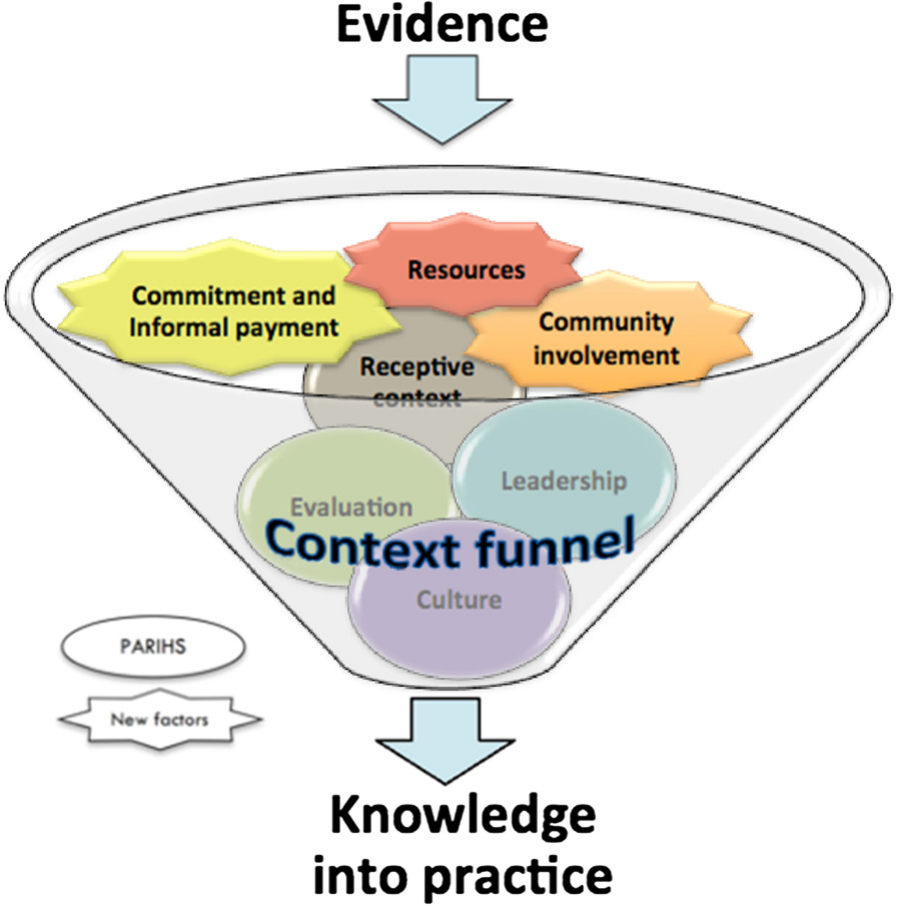
PARIHS sub-elements and additionally identified factors.

The great majority of sub-elements and concepts in the context cornerstone of PARIHS were found also to be relevant in this low-income setting. There were also additional factors in the organizational context that were perceived to influence KT, such as commitment and informal payment and community involvement (Figure 2).

In this study, respondents described commitment as the individual’s devotion to the organization. The lack of commitment was often brought up as a barrier to KT, because uncommitted health workers were perceived as less likely to change. Commitment was at first seen as reflecting ‘low-culture’ where health workers did not share the values of the organization or as ‘low-receptiveness to change’ as respondents described committed health workers to be more prone to change. However, because commitment in this study is reflecting an ‘individual’s devotion to the organization he/she belongs to, we opted to present it separately following discussions in the research team and with peer de-briefers. To increase the understanding of organizational context, we believe commitment needs more attention. We found that the shortage of human resources undermines commitment, similar to findings by McAuliffe *et al*., who explored the work environment of mid-level healthcare providers in Malawi [40]. They found that the shortage of human resources correlated with emotional exhaustion, job dissatisfaction, dissatisfaction with one’s profession, and thinking about leaving one’s job.

Another factor identified in this study, which partly related to low salary and lack of commitment, was the existence of informal payment, which Lewis defines as ‘payments to individual and institutional providers, in kind or in cash, that are made outside official payment channels or are purchases meant to be covered by the healthcare system’ [41]. Respondents brought up the fact that health workers were selling drugs that should be available for free to patients, thereby falling short in securing resources for patients who needed them. Furthermore, health workers had to provide informal payment to acquire new positions. Similar findings have been reported from many settings, and informal payments are commonly reported as hindering development in low-income settings [42,43]. A recent Tanzanian study indicates that health workers create artificial shortages of drugs and supplies and deliberately lower the quality of service in order to collect extra payments from patients [44]. The impact of corruption has been reported as influencing the implementation of health sector reforms, and also as having an additional demoralizing effect on health workers, thereby having a negative influence on health services in general [45,46]. Informal payment is likely to be a key factor influencing not only routine health service delivery, but also the implementation of EBP. Thus, we consider that commitment and informal payment should be part of an assessment tool for low-income settings.

Our study clearly indicated that lack of resources is a hindrance to KT in the current setting. Resources brought up by respondents referred to available assets that would enable the functioning of their healthcare units and could be divided into four types: human resources; space; communication and transport; and medicine, equipment and other supplies. Availability of resources is also proposed as one component of a receptive context by the PARIHS team [22]. The PARIHS team claim that the relationship between available resources and implementation of EBPs is not straightforward, and that increased resources need to be appropriately allocated and managed in order to influence the implementation process positively. In addition, the PARIHS team also point out that ‘the focus on resources should not be at the expense of deeper issues such as relationships, cultures, and ways of working’ [20]. Our findings underline that resources are of major importance in a setting that is suffering from the lack thereof. Ovretveit *et al*. [47] suggest that decision makers should investigate those resources that are needed prior to the implementation of new interventions, in order to avoid trying to implement strategies that require resources that cannot be mobilized. Such an investigation also links to the definition of evaluation provided in the public health dictionary [30] that states that one important reason for evaluating health services is to answer questions about costs in relation to benefits. While resources are an essential aspect of healthcare improvement in large parts of the world, we think that ‘resources’ should be considered as a freestanding sub-element of context in the PARIHS framework when applied in low-income settings.

The relevance of a supportive culture in order for effective KT to take place was obvious. Respondents brought up that sharing new knowledge while working was of fundamental importance, perhaps reflecting the lack of organizational slack, leading to few opportunities to share knowledge. For the sharing of knowledge to occur, the importance of good teamwork was emphasized. The current findings further indicate that interdisciplinary teamwork is important, an element that is underlined by results in other studies as leading to fewer errors and shorter delays [48-51]. Our findings are also congruent with those of a recent study in Kenya, where the lack of interdisciplinary teamwork was identified as a barrier to successful implementation of guidelines [52]. The relevance of formal meetings to discuss the provision of care was evident in the interviews and has been shown to lead to improved practice elsewhere [53,54]. Also consistent with our findings is the importance of approaching and involving lower-level health workers in the evaluation and planning of health services [22]. Our findings indicate that the current definition and components of culture according to the PARIHS framework (as described in Figure 1) are also of relevance in the current setting.

Leadership was perceived as being important for promoting effective KT. In particular, midwives working in health centers were dependent on their leaders to acquire new knowledge and were therefore much affected by their absence. In general, respondents described good leadership for KT in words that could be linked to the concept of transformational leadership as described in the PARIHS framework [20]. Effective leadership gives rise to clear roles and effective teamwork and organizational structures [17]. In our study, there was a perceived lack of leadership in rural and isolated health centers that is likely to have a major negative impact on KT. Absenteeism of leaders in lower-level health centers is a common phenomenon in many low-income settings, and falls under the broad term of ‘quiet corruption,’ defined by the World Bank as: ‘when public servants fail to deliver services or inputs that have been paid for by the government’ [42]. McPake *et al*. studied absenteeism in Uganda, and found that poor quality of healthcare services created a downward spiral of underutilization of public health facilities where lower demand for services led to even lower staff attendance and shorter opening hours [55]. Similar to our findings, Manongi and co-workers found that lack of supervision and feedback left health workers in lower-level health centers feeling unsupported and undervalued [56]. In contrast, findings in a Kenyan study [57] suggest that supportive leadership might foster a supportive culture and enable good working relationships between different types of healthcare providers. In 2007, Snowden and Boon presented four types of leadership, suited for four types of contexts [58]. Simple systems, being relatively stable with clear cause-and-effect relationships, are suited for traditional leadership styles in terms of command and control, delegation of tasks to well defined roles, organized structures, and discrete evaluations. As systems get more complicated, there is an increased demand for the leaders to rely on facilitation and empowerment of others [58]. Our findings do indicate that the health system under study is complicated, requiring leaders that model the openness and reflection needed to communicate the vision of the organization, providing the support needed to lead others towards it [59]. These leadership qualities reflect transformational leadership as described in the PARIHS framework [20].

We found that community involvement can work as a driver to allow KT to be part of an evaluation system. The relevance of such community involvement as a component influencing the KT process in low-income settings is likely to be high because consumer demand creates a need for the local health system to improve. There are currently numerous ongoing trials in low-income settings studying the link between the community and healthcare providers by evaluating community involvement in the health systems. Such efforts have proven to be effective in some studies [60-62]. However, changing the behaviour of community members in seeking healthcare is a slow process. A recent review from the World Bank identified community ownership—that is, to support communities to take part in, contribute to, and be accountable for an intervention—as one successful approach to KT [63]. Similarly, Du Mortier and Arpagaus found that involving community members in creating quality of care indicators helped communities to take ownership of healthcare evaluation and improvement [64]. Taking these findings into consideration, we suggest community involvement should also be part of the assessment of organizational context in low-income settings. In addition to the identified importance of community involvement, formal evaluation and feedback were also perceived as important in the current study setting. Participants thought it was important to evaluate healthcare performance in order to initiate change. In terms of receiving feedback on performance, the findings are in line with the proposed components of the evaluation sub-element (Figure 1). The PARIHS framework considers evaluation as a monitoring and feedback strategy, using multiple sources and methods, to improve the provision of healthcare [17]. Coherent with our findings and PARIHS, feedback and recognition of health workers has previously been shown to influence KT positively [22,63,65,66]. We believe that KT would be further strengthened if the Evaluation cornerstone of the PARIHS framework clearly included end-users engagement and evaluation of health services.

There is much evidence to show those interventions that should be implemented in order to reduce the burden of perinatal mortality and, increasingly, researchers argue that there is as great need to understand the social and system context as epidemiology when designing healthcare programmes to improve perinatal health outcomes [67,68]. The World Health Organization (WHO) proposes that the health system is composed of six interconnected building blocks; governance, information, financing, service delivery, human resources, and medicines and technologies [69]. Both PARIHS and the WHO health system building blocks enable a structured description of the health system, thereby going beyond seeing the health system as a ‘black box.’ Our findings identify a number of structures in the local healthcare system relating to these two theoretical models and could be a point of departure to develop a context assessment tool. Overall, the findings of our study are similar to those of one conducted in Kenya [52] that identified ten barriers to the implementation of guidelines, including: poor communication and teamwork; organizational constraints and limited resources; lack of recognition and appreciation of good work; absence of perceived benefits linked to adoption of new practices; and lack of motivation. These similarities, and the links to findings in other low-income countries, and not least the concordance with the PARIHS framework, suggest that the factors identified might be of general importance, and not only of relevance in the currently investigated context.

### Methodological considerations

Although we reached saturation amongst the study participants we targeted, one limitation was the small sample. Because this study is a part of a larger study aimed at developing a context assessment tool for low- and middle-income settings, additional efforts of this kind from other settings will generate more insights into the transferability of these findings. This study explored midwives’ and managers’ perception of organizational characteristics influencing implementation of new knowledge in their place of work, that is, the ‘internal context.’ However, the internal context is embedded in a larger health system that further influences implementation; the ‘outer context’ was, however, not included in the scope of the current study.

We did not find any differences in the managerial versus midwives’ responses, and we believe that this merely reflects that the managers are also health workers who are, and have been, subjected to changes. Areas in which the research group were not in agreement at first included whether ‘commitment’ should be seen as an ‘individual’ factor, or if it affects the ‘organizational’ in such a way that it should, in fact, also be included in a future assessment tool. Discussions amongst researchers and peer de-briefers were undertaken in iterative conference calls and via email and we finally agreed to present ‘commitment’ as a factor of importance to include in an organization context assessment tool.

Exploration of an existing framework in a new context was challenging in terms of how to handle different concepts. Although ‘resources’ is one component of the sub-element ‘receptive context’ in the PARIHS framework, we found that it was of such weight in this setting that it must be given more attention and definitely not be left out as seen in some descriptions of the framework [32,33,70]. In a later version of the framework, allocation of resources even appear under the sub-element ‘culture’ [71]. In order to both take into account how the framework is presented in different ways but primarily for staying true to our findings, we decided to present ‘resources’ as a stand-alone factor.

## Conclusions

Improved understanding of the organizational context will promote KT, not only in high-income countries, but also in low-income countries. The components of organizational context as suggested by the PARIHS framework appear also to be relevant in a low-income setting like Uganda. In addition, resources, commitment and informal payment, and community involvement should be considered as important components for developing context assessment tools for low-income settings.

## Abbreviations

AB: Anna Bergström; EBP: Evidence-based practices; FDG: Focus group discussion; KT: Knowledge translation; PARIHS: Promoting Action on Research Implementation in Health Services; SN: Sarah Namusoko.

## Competing interests

The authors declare that they have no competing interests.

## Authors’ contributions

This study was designed by AB, LW, SP and PW. Data collection and a first review of findings were performed by AB and SN. Verbatim transcription was undertaken by AB, who was responsible for the data analysis. The draft manuscript was written by AB and LW. All authors have read and approved the final manuscript.

## Supplementary Material

Additional file 1 Data collection guide for context study. Click here for file
