# Supplementary material for: Knowledge translation in Uganda: a qualitative study of Ugandan midwives’ and managers’ perceived relevance of the sub-elements of the context cornerstone in the PARIHS framework
Source: Implement Sci. 2012 Dec 3;7:117. doi: 10.1186/1748-5908-7-117 (PMC3538529; doi:10.1186/1748-5908-7-117)
Supplement: Additional file 1 — Data collection guide for context study. [file 1748-5908-7-117-S1.docx]

**DATA COLLECTION GUIDE FOR CONTEXT STUDY**

**CHANGING PRACTICE**

So, I would like to understand how the organization of health care, the organizational context, influences the uptake of new knowledge into practice.

First I would like us to understand how practices are being changed within health care.

Can you give me an example of a change in practice that has recently occurred where you work?

**Q:** If you think about the changes that you have spoken of now, how were they initiated?

*Suggested probes:*

- Top up
- Top down

**Q:** What influences the process of changing practice?

*Suggested probe:*

- What makes it successful/unsuccessful?

**Q:** How willing are people to change practices?

**Q:** What do you think might increase your use of new knowledge?

**CULTURE: “the way we do things”**

Now I would like us to discuss the culture in your place of work.

**Q:** To what extent do you feel that you are being supported by your workmates/leaders in the organization to professional development?

**Q:** How important is it for you to develop professionally?

*Suggested probes:*

- Why? How? Examples?

**Q:** You’re a midwife, how clear is your work description?

*Suggested probes:*

- Do you know when you are doing a job of another cadre? Does it ever happen?
- Examples?
- Who decides your responsibilities?

**Q:** If you have done a good job, how is it recognized?

*Suggested probes:*

- Is recognition important? By whom? How?

**EVALUATION AND FEEDBACK**

I would now like us to focus on how gaps in knowledge are identified.

**Q:** How are knowledge-gaps identified in your place of work?

*Suggested probes:*

- Poor patient outcome?
- Informal discussions?

**Q:** When you have identified such a gap, what action is taken to improve identified problems?

*Suggested probes:*

- Who is involved in the process?
- Action planning (deciding how to solve and issue, who is to solve it and by when)?

**Q:** What type of feedback do you get regarding how you perform your professional work?

*Suggested probes:*

- Is feedback important? By whom? How?
- Positive/negative feedback.

**LEADERSHIP**

I would like us to focus on leadership.

**Q:** If you think about your nearest boss, does he/she

1. Support you when trying to improve clinical practice?
2. Pressure you to improve clinical practice?

**Q:** On a wider perspective, what type of support/pressure do you have when it comes to improve clinical practice?

**Q:** Describe the leadership in your workplace

*Suggested probes:*

- In the facility
- In the ward
- During a shift

**Q:** How open is the hierarchy in this unit for you to discuss problems with your leaders (are they approachable)?
